# Real-world approach to managing dysgeusia following the use of esketamine nasal spray: a case report

**DOI:** 10.1186/s12991-020-00262-x

**Published:** 2020-02-26

**Authors:** Nicholas A. Bossaller, Richard C. Shelton

**Affiliations:** Department of Psychiatry, University of Alabama School of Medicine, Birmingham, AL 35294 USA

**Keywords:** Esketamine nasal spray, Dysgeusia, Nausea, Vomiting, Antiemetic

## Abstract

**Background:**

Patients with depression who are treated with esketamine nasal spray may commonly experience dysgeusia (bad/metallic/bitter taste) and related side effects such as nausea and vomiting. While pretreatment with antiemetics can mitigate or prevent nausea and vomiting, it may not address dysgeusia as a contributing factor. Alternative interventions could help to manage vomiting due to dysgeusia following administration of esketamine nasal spray in those patients who are affected.

**Case presentation:**

A 40-year-old man presented to the emergency department with depression and started treatment with an oral antidepressant. After providing informed consent to participate in a clinical trial evaluating the efficacy and safety of esketamine for major depressive disorder with active suicidal ideation with intent, he received 84 mg of esketamine nasal spray twice per week for 4 weeks. On the first 2 days of esketamine administration, the patient reported dysgeusia lasting several hours and intermittent retching lasting approximately 20 min. The patient was then given a fruit punch–flavored powdered drink (Crystal Light Fruit Punch™) approximately 25 min after nasal spray administration during the study period. The use of a fruit punch drink resulted in notable improvement of dysgeusia and associated vomiting, with time to resolution occurring within 30 min of the report of the adverse event.

**Conclusions:**

A fruit punch–flavored powdered drink mix taken shortly after administration of esketamine nasal spray may rapidly manage and prevent vomiting due to dysgeusia.

## Background

Patients with treatment-resistant depression who use esketamine nasal spray may commonly experience some unique side effects. To date, 19% of patients treated with esketamine nasal spray plus an oral antidepressant in clinical trials have experienced dysgeusia (bad/metallic/bitter taste), as well as potentially related side effects including nausea (28%) and vomiting (9%) [1]. At least one case has been reported of a patient discontinuing treatment because of nausea [2]. A potential solution to mitigate or prevent nausea and vomiting is to pretreat using an antiemetic, such as ondansetron; however, this may not address dysgeusia as a contributing factor.

We describe here the management of a patient affected by vomiting due to dysgeusia after receiving esketamine nasal spray. To our knowledge, this strategy has not been reported in the literature and is a low-cost, low-risk, and simple solution to a common problem using an easily sourced product.

## Case presentation

A 40-year-old man presented to the University of Alabama at Birmingham Medical Center emergency department with depression. He was admitted to the hospital and started on oral fluoxetine 20 mg daily, titrating to 40 mg daily after the initial 3 days. The patient then gave written informed consent to participate in an investigational study examining rapid reduction of depressive symptoms in patients with major depressive disorder with active suicidal ideation with intent. At the time of writing this case report, esketamine nasal spray has been approved only for the management of treatment-resistant depression (TRD). He met the trial eligibility criteria and was randomized to esketamine nasal spray, which was initiated on day 1 at a dose of 84 mg administered using 3 nasal spray devices (28 mg/device) twice per week for 4 weeks. After the initial dose, the patient reported dysgeusia almost immediately and began intermittently retching, which delayed the administration of the subsequent dose. He continued retching intermittently for 14 min, and the bad taste persisted for approximately 3 h.

After the third dose was administered on the first day, the patient was encouraged to rinse out his mouth with water and spit it out without swallowing. However, the patient retched within 2 min and the bad taste persisted for several hours. On the second dosing day, rinsing with water was attempted again, with a similar lack of effect. After 20 min of intermittent retching, a nurse suggested a fruit punch–flavored powdered drink (Crystal Light Fruit Punch) mixed with water to mask the taste, which the patient drank 25 min after the initial spray. The bad taste resolved within 5 min (30 min after the report of the adverse event). During the third dosing day, dysgeusia was reported again, but after drinking the punch-flavored beverage, the time to resolution decreased to 24 min after the adverse event was reported. This strategy was employed for all subsequent doses, and the patient reported only one more instance of dysgeusia on the final day of dosing. Again, time to resolution was within 30 min of the report of the adverse event. A punch-flavored drink has been used successfully to reverse or prevent dysgeusia with other patients at our center.

## Discussion

Esketamine nasal spray is a noncompetitive *N*-methyl D-aspartate (NMDA) receptor antagonist [1]; thus, its primary antidepressant activity is believed not to involve direct inhibition of serotonin, norepinephrine, or dopamine reuptake [3]. Nausea, retching, and vomiting are common side effects associated with the use of esketamine nasal spray. Dysgeusia may contribute to or cause nausea and vomiting, and patients treated with esketamine nasal spray plus an oral antidepressant in clinical trials have reported dysgeusia as a side effect. Antiemetics such as ondansetron [4] may reverse or prevent nausea and vomiting related to the direct pharmacological effects of esketamine and may be considered as a prophylactic treatment in patients who continue to experience nausea and vomiting with treatment. At our center, antiemetics are generally administered to patients who have retched for ≥ 10 min or are given as a pretreatment when nausea and vomiting occur as a recurrent adverse event. Ondansetron may also be effective in restoring appetite in patients undergoing cancer chemotherapy who experience an altered taste of food (a type of dysgeusia) [5]. However, it is unclear if it is effective in treating nausea due to drug-related dysgeusia. We hypothesize that the mechanism of dysgeusia associated with esketamine nasal spray administration relates to postnasal dripping of residual spray into the oral cavity and contact with the posterior tongue and taste buds, triggering the taste receptor cells. In the pivotal studies of esketamine nasal spray, a bittering agent was added to the placebo nasal spray in an effort to maintain the blinding; dysgeusia was reported by 14% (*n* = 30) of patients in the placebo arm compared with 19% (*n* = 60) in the esketamine nasal spray arm [1]. In the present case, the use of a fruit punch–flavored powdered drink mix in water reversed dysgeusia associated with administration of esketamine nasal spray. This strategy is commonly used by nurses in our facility with other medications that can cause dysgeusia, nausea, and vomiting. The exact physiological mechanism behind this strategy is unknown, although we believe the beverage masks the taste of esketamine nasal spray. Fruit punch–flavored powdered drink mixes are available in small packets that can be easily stored or transported, making it a safe, convenient, and easily accessible method for managing these side effects.

## Conclusions

Clinicians may consider implementing a simple strategy to mitigate one of the more common side effects reported with esketamine. Namely, clinicians may consider use of a fruit punch–flavored powdered drink mix taken shortly after administration of esketamine nasal spray, which may rapidly manage and prevent vomiting due to dysgeusia.

## Data Availability

The data sharing policy of Janssen Pharmaceutical Companies of Johnson & Johnson is available at https://www.janssen.com/clinical-trials/transparency. As noted on this site, requests for access to the study data can be submitted through the Yale Open Data Access (YODA) Project site at https://yoda.yale.edu.

## References

[1] Janssen Pharmaceuticals. SPRAVATO™ (esketamine) nasal spray. 2019. https://www.janssenlabels.com/package-insert/product-monograph/prescribing-information/SPRAVATO-pi.pdf. Accessed Feb 2020.

[2] Popova V, Daly EJ, Trivedi M, Cooper K, Lane R, Lim P (2019). Efficacy and safety of flexibly dosed esketamine nasal spray combined with a newly initiated oral antidepressant in treatment-resistant depression: a randomized double-blind active-controlled study. Am J Psychiatry.

[3] Krystal JH, Sanacora G, Blumberg H, Anand A, Charney DS, Marek G (2002). Glutamate and GABA systems as targets for novel antidepressant and mood-stabilizing treatments. Mol Psychiatry.

[4] GSK. Ondasetron prescribing information. 2011. https://www.accessdata.fda.gov/drugsatfda_docs/label/2011/020103s030,020605s014,020781s014lbl.pdf. Accessed Feb 2020.

[5] Edelman MJ, Gandara DR, Meyers FJ, Ishii R, O'Mahony M, Uhrich M (1999). Serotonergic blockade in the treatment of the cancer anorexia–cachexia syndrome. Cancer.

